# Differential modulation of microglia superoxide anion and thromboxane B_2 _generation by the marine manzamines

**DOI:** 10.1186/1471-2210-5-6

**Published:** 2005-03-11

**Authors:** Alejandro MS Mayer, Mary L Hall, Sean M Lynch, Sarath P Gunasekera, Susan H Sennett, Shirley A Pomponi

**Affiliations:** 1 Department of Pharmacology, Chicago College of Osteopathic Medicine, Midwestern University, 555 31st Street, Downers Grove, Illinois 60515, USA; 2 Department of Pharmacology, Chicago College of Osteopathic Medicine, Midwestern University, 555 31st Street, Downers Grove, Illinois 60515, USA; 3 Department of Biochemistry, Chicago College of Osteopathic Medicine, Midwestern University, 555 31st Street, Downers Grove, Illinois 60515, USA; 4 Division of Biomedical Marine Research, Harbor Branch Oceanographic Institution, Inc. 5600 US # 1 North, Fort Pierce, Florida 34946, USA; 5 Division of Biomedical Marine Research, Harbor Branch Oceanographic Institution, Inc. 5600 US # 1 North, Fort Pierce, Florida 34946, USA; 6 Division of Biomedical Marine Research, Harbor Branch Oceanographic Institution, Inc. 5600 US # 1 North, Fort Pierce, Florida 34946, USA

## Abstract

**Background:**

Thromboxane B_2 _(TXB_2_) and superoxide anion (O_2_^-^) are neuroinflammatory mediators that appear to be involved in the pathogenesis of several neurodegenerative diseases. Because activated-microglia are the main source of TXB_2 _and O_2_^- ^in these disorders, modulation of their synthesis has been hypothesized as a potential therapeutic approach for neuroinflammatory disorders. Marine natural products have become a source of novel agents that modulate eicosanoids and O_2_^- ^generation from activated murine and human leukocytes. With the exception of manzamine C, all other manzamines tested are characterized by a complex pentacyclic diamine linked to C-1 of the β-carboline moiety. These marine-derived alkaloids have been reported to possess a diverse range of bioactivities including anticancer, immunostimulatory, insecticidal, antibacterial, antimalarial and antituberculosis activities. The purpose of this investigation was to conduct a structure-activity relationship study with manzamines (MZ) A, B, C, D, E and F on agonist-stimulated release of TXB_2 _and O_2_^- ^from *E. coli *LPS-activated rat neonatal microglia *in vitro*.

**Results:**

The manzamines differentially attenuated PMA (phorbol 12-myristate 13-acetate)-stimulated TXB_2 _generation in the following order of decreasing potency: MZA (IC_50 _<0.016 μM) >MZD (IC_50 _= 0.23 μM) >MZB (IC_50 _= 1.6 μM) >MZC (IC_50 _= 2.98 μM) >MZE and F (IC_50 _>10 μM). In contrast, there was less effect on OPZ (opsonized zymosan)-stimulated TXB_2 _generation: MZB (IC_50 _= 1.44 μM) >MZA (IC_50 _= 3.16 μM) >MZC (IC_50 _= 3.34 μM) >MZD, MZE and MZF (IC_50 _>10 μM). Similarly, PMA-stimulated O_2_^- ^generation was affected differentially as follows: MZD (apparent IC_50_<0.1 μM) >MZA (IC_50 _= 0.1 μM) >MZB (IC_50 _= 3.16 μM) >MZC (IC_50 _= 3.43 μM) >MZE and MZF (IC_50 _>10 μM). In contrast, OPZ-stimulated O_2_^- ^generation was minimally affected: MZB (IC_50 _= 4.17 μM) >MZC (IC_50 _= 9.3 μM) >MZA, MZD, MZE and MZF (IC_50 _> 10 μM). From the structure-activity relationship perspective, contributing factors to the observed differential bioactivity on TXB_2 _and O_2_^- ^generation are the solubility or ionic forms of MZA and D as well as changes such as saturation or oxidation of the β carboline or 8-membered amine ring. In contrast, the fused 13-membered macrocyclic and isoquinoline ring system, and any substitutions in these rings would not appear to be factors contributing to bioactivity.

**Conclusion:**

To our knowledge, this is the first experimental study that demonstrates that MZA, at *in vitro *concentrations that are non toxic to *E. coli *LPS-activated rat neonatal microglia, potently modulates PMA-stimulated TXB_2 _and O_2_^- ^generation. MZA may thus be a lead candidate for the development of novel therapeutic agents for the modulation of TXB_2 _and O_2_^- ^release in neuroinflammatory diseases. Marine natural products provide a novel and rich source of chemical diversity that can contribute to the design and development of new and potentially useful anti-inflammatory agents to treat neurodegenerative diseases.

## Background

The hallmark of brain inflammation is the activation of glia, particularly microglia, the resident immune cells of the brain [1]. Microglia activation in brain pathologies, as caused by infectious diseases, inflammation, trauma, brain tumors, ischemia and AIDS, may result in neuronal injury and ultimately neurodegeneration [1]. Similar to other tissue macrophages, when microglia become activated they release potentially neurotoxic mediators [2], followed by sublethal and lethal injury to the central nervous system. The two different phenotypic forms of microglia, namely the activated but nonphagocytic microglia found in inflammatory pathologies and the reactive or phagocytic microglia present in trauma, infection and neuronal degeneration, appear to have the capacity to express cell-surface receptors and release mediators of inflammation, such as cytokines, coagulation factors, complement factors, proteases, nitric oxide, eicosanoids and reactive oxygen species [2].

Over the last three decades, the marine environment has been demonstrated to be a source of novel therapeutic agents, many of which have anti-inflammatory properties [3]. We have previously shown that selected marine natural products modulate eicosanoids [4,5] and O_2_^- ^generation from activated rat [6] and human neutrophils [7], as well as liver [8] and alveolar macrophages [9]. Based on these observations we hypothesized that selected marine natural products might potentially attenuate activated brain microglia [2]. Since the discovery by Sakai and Higa that the marine sponge-derived manzamine A (MZA) had potent antitumor activity [10], there has been a sustained interest in the chemistry [11] as well as the pharmacology of the manzamines, a class of β-carboline marine-derived alkaloids. More than 40 manzamine-type alkaloids have been isolated from 9 different genera of marine sponges from the Indian and Pacific Oceans, and in addition to the antitumor activity [10], manzamines have been shown to be immunostimulatory [12], insecticidal [13], antibacterial[13], antimalarial [14], antiparasitic [15], antiviral [16] and to possess antituberculosis activity [17].

In preliminary communications we have reported that MZA, isolated from the Okinawan marine sponge *Haliclona *sp., potently inhibited TXB_2 _and O_2_^- ^generation by activated rat neonatal microglia while showing very low concomitant toxicity [18-20]. We now extend these previous communications by reporting the results of a structure-activity relationship study with manzamines A, B, C, D, E and F on agonist-stimulated release of O_2_^- ^and TXB_2 _from LPS-activated rat neonatal microglia.

## Results

### Effect of manzamine A on LPS-activated neonatal brain microglia TXB_2_, O_2_^- ^and LDH release

**Figure 1:**
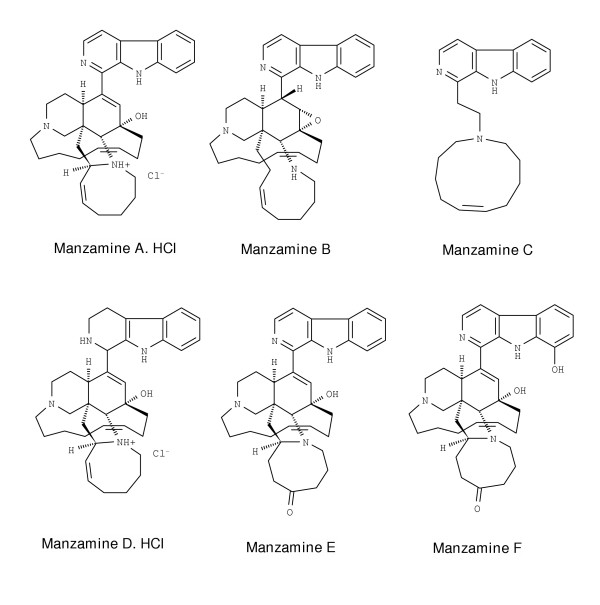
**The chemical structures of manzamines A, B, C, D, E and F. **Manzamines are indole-derived alkaloids isolated from the marine sponges *Haliclona sp*. [10], *Amphimedon sp*. [66] and *Xestospongia sp*. [10,67]. Molecular weights are respectively = 585.2, 550.8, 347.5, 591.3, 564.7, 580.8.

**Figure 2:**
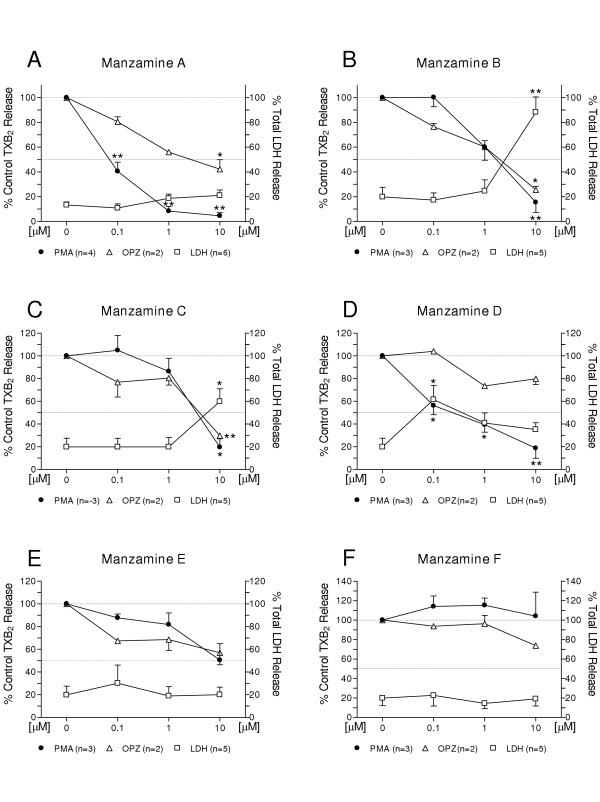
**Differential effects of manzamines A, B, C, D, E and F on PMA and OPZ-stimulated TXB_2 _generation by LPS-activated rat neonatal microglia**. Rat neonatal microglia (200,000 cells/well) were activated with LPS (0.3 ng/mL) for 17 hours. Manzamines were added 15 min before stimulation with either PMA (1 μM) or OPZ (0.5 mg/mL). After 70 min, agonist-triggered TXB_2 _was measured as described in *Materials and Methods*. LDH release, indicator of cytotoxicity, was determined as described in *Materials and Methods*. Data are expressed as percentage of control TXB_2 _release triggered by either PMA (MZA: 1,423 ± 439 pg TXB_2 _/70 min; MZB, C, D, E, F: 3,279 ± 281 pg TXB_2 _/70 min), or OPZ (4,504 ± 308 pg TXB_2 _/70 min). Data show mean ± SEM of indicated number (n) of experiments. * P < 0.05, ** P < 0.01.

**Figure 3:**
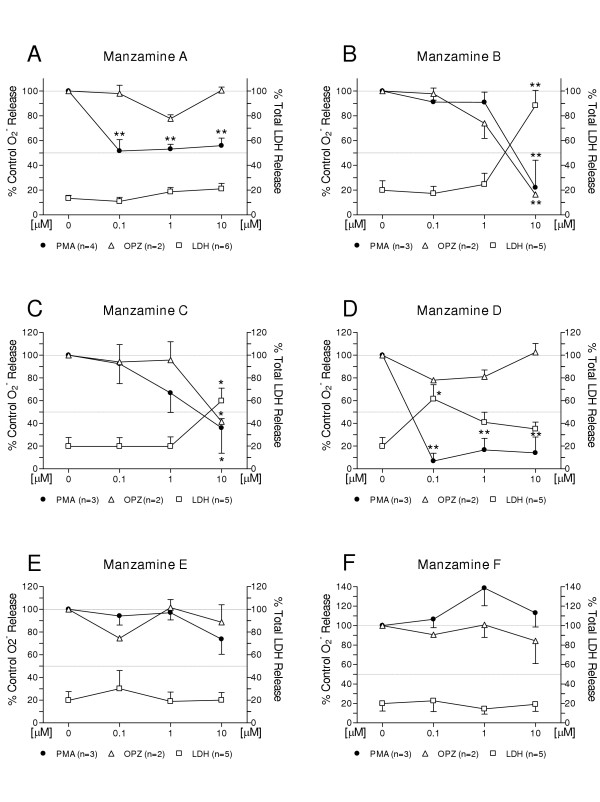
**Differential effects of manzamines A, B, C, D, E and F on PMA and OPZ-stimulated O_2_^- ^generation by LPS-activated rat neonatal microglia**. Rat neonatal microglia (200,000 cells/well) were activated with LPS (0.3 ng/mL) for 17 hours. Manzamines were added 15 min before stimulation with either PMA (1 μM) or OPZ (0.5 mg/mL). After 70 min, agonist-triggered O_2_^- ^release was determined as described in *Materials and Methods*. LDH release, indicator of cytotoxicity, was measured as described in *Materials and Methods*. Data are expressed as percentage of control O_2_^- ^release triggered by PMA (MZA: 13.8 ± 1.9 nmoles O_2_^-^/70 min; MZB, C, D, E, F: 10.8 ± 0.6 nmoles O_2_^-^/70 min), or OPZ (9.4 ± 0.7 nmoles O_2_^- ^/70 min). Data show mean ± SEM of indicated number (n) of experiments. * P < 0.05, ** P < 0.01.

As shown in Fig. 1, MZA has a pentacyclic diamine group attached to the β-carboline moiety and it was tested as its hydrochloride salt. As is shown in Fig. 2A, MZA potently inhibited PMA-stimulated TXB_2 _generation (IC_50 _= 0.016 μM), with a maximum 95.5 % inhibition observed at 10 μM (MZA vs. vehicle, respectively 78.3 ± 45 vs. 1,413 ± 439 pg of TXB_2 _per 200,000 microglia per 70 min, P < 0.01, n = 4). Furthermore, as depicted in Fig. 3A, MZA inhibited PMA-stimulated O_2_^- ^generation with an apparent IC_50 _= 0.1 μM (MZA vs. vehicle, respectively 7.5 ± 1.8 vs.13.8 ± 1.9 nmol of O_2_^- ^per 200,000 microglia per 70 min, P < 0.01, n = 4). Significantly, increasing MZA concentrations to 10 μM resulted in O_2_^- ^inhibition of 55.9 ± 6.1 %, P < 0.01, n = 4.

In contrast, as shown in Fig. 2A, the effect of MZA on OPZ-stimulated TXB_2 _generation was weaker (apparent IC_50 _= 3.16 μM), with a maximum 58 % inhibition at 10 μM (MZA vs. vehicle, respectively 1,927 ± 474 vs. 4,504 ± 308 pg of TXB_2 _per 200,000 microglia per 70 min, P < 0.05, n = 2). Similarly, as depicted in Fig 3A, MZA did not appear to affect OPZ-stimulated O_2_^- ^generation even at 10 μM (MZA vs. vehicle, respectively 9.5 ± 1 vs. 9.4 ± 0.7 O_2_^- ^nmol per 200,000 microglia per 70 min, P > 0.05, n = 2).

As shown in Fig. 2A and 3A, the cytotoxicity of MZA to neonatal brain microglia measured as LDH release was not significantly different from controls even at 10 μM (MZA vs. vehicle, respectively 21.3 ± 7 % vs. 13.9 ± 3.7 % of total LDH released by 0.1 % Triton X-100 treated-microglia, n = 6, P = 0.1735). This data suggests that the effect of MZA on both PMA-stimulated TXB_2 _and O_2_^- ^may be of a pharmacological nature.

### Effect of manzamine B on LPS-activated neonatal brain microglia TXB_2_, O_2_^- ^and LDH release

MZB differs from MZA in having a tetracyclic diamine complex and an epoxide ring system (Fig. 1). As shown in Fig. 2B, MZB which was tested as a free base, was less potent than MZA in affecting PMA-stimulated TXB_2 _generation (IC_50 _= 1.6 μM). MZB 10 μM reduced TXB_2 _release to 15.5 % of control (MZB vs. vehicle, respectively 546.3 ± 281 vs. 3,279 ± 281 pg of TXB_2 _per 200,000 microglia per 70 min, P < 0.01, n = 3). Similarly, as depicted in Fig. 3B, MZB was less potent than MZA in affecting PMA-stimulated O_2_^- ^generation (IC_50 _= 3.16 μM). MZB 10 μM reduced O_2_^- ^release to 20 % of control (MZB vs. vehicle, respectively 2.14 ± 2.14 vs. 10.8 ± 0.6 nmol of O_2_^- ^per 200,000 microglia per 70 min, P < 0.01, n = 3).

As shown in Fig. 2B, MZB affected OPZ-stimulated TXB_2 _(IC_50 _= 1.44 μM) more than MZA. MZB 10 μM reduced TXB_2 _generation to 25.6 % of control (MZB vs. vehicle, respectively 1,160 ± 207 vs. 4,504 ± 308 pg of TXB_2 _per 200,000 microglia per 70 min, P < 0.05, n = 2). Furthermore, as shown in Fig. 3B, MZB reduced OPZ-stimulated O_2_^- ^generation (IC_50 _= 4.17 μM) more than MZA. MZB 10 μM reduced O_2_^- ^generation to 16.5 % of control (MZB vs. vehicle, respectively 1.5 ± 0.7 vs. 9.4 ± 0.7 O_2_^- ^nmol per 200,000 microglia per 70 min, P < 0.01, n = 2).

As shown in Fig. 2B and 3B, in contrast to MZA, MZB was cytotoxic to neonatal brain microglia at concentrations above 1 μM. In fact, considerable LDH release was observed at 10 μM (88.3 ± 12 % of total LDH released by 0.1 % Triton X-100 treated-microglia, n = 5, P < 0.01). Taken together, these data suggest that the reduction of both O_2_^- ^and TXB_2 _generation resulted from both pharmacological and toxic effects of MZB on LPS-activated microglia cells.

### Effect of manzamine C on LPS-activated neonatal brain microglia TXB_2_, O_2_^- ^and LDH release

MZC differs from MZA in having a monocyclic amine ring attached to the β-carboline moiety (Fig. 1). As shown in Fig. 2C, MZC which was tested as a free base, was less potent than MZA in affecting PMA-stimulated TXB_2 _generation (IC_50 _= 2.98 μM). MZC 10 μM reduced TXB_2_release to 19.6 % of control (MZC vs. vehicle, respectively 677 ± 293 vs. 3,279 ± 281 pg of TXB_2 _per 200,000 microglia per 70 min, P < 0.01, n = 3). Similarly, as depicted in Fig. 3C, MZC was less potent than MZA in affecting PMA-stimulated O_2_^- ^generation (apparent IC_50 _= 3.43 μM). MZC 10 μM reduced O_2_^- ^release to 36.1 % of control (MZC vs. vehicle, respectively 3.7 ± 2.2 vs.10.8 ± 0.6 nmol of O_2_^- ^per 200,000 microglia per 70 min, P < 0.05, n = 3).

As shown in Fig. 2C, MZC affected OPZ-stimulated TXB_2 _(IC_50 _= 3.34 μM), similar to MZA. MZC 10 μM reduced TXB_2 _generation to 29.8 % of control (MZC vs. vehicle, respectively 1,345 ± 160 vs. 4,504 ± 308 pg of TXB_2 _per 200,000 microglia per 70 min, P < 0.05 n = 2). Furthermore, as depicted in Fig. 3C, MZC reduced OPZ-stimulated O_2_^- ^generation (apparent IC_50 _= 9.3 μM), higher than MZA. MZC 10 μM reduced O_2_^- ^generation to 41.6 % of control (MZC vs. vehicle, respectively 3.9 ± 0.3 vs. 9.4 ± 0.7 O_2_^- ^nmol per 200,000 microglia per 70 min, P < 0.05, n = 2).

As shown in Fig. 2C and 3C, and in contrast to MZA, MZC was cytotoxic to neonatal brain microglia though not as much as MZB. Substantial LDH release was observed at 10 μM (59.8 ± 11 % of total LDH released by 0.1 % Triton X-100 treated-microglia, n = 5, P < 0.05). In summary, similar to MZB, the data suggests that the reduction of both O_2_^- ^and TXB_2 _generation resulted from both pharmacological and toxic effects of MZC on LPS-activated microglia cells.

### Effect of manzamine D on LPS-activated neonatal brain microglia TXB_2_, O_2_^- ^and LDH release

MZD differs from MZA in having a tetrahydrocarboline group in the molecule (Fig. 1). As shown in Fig. 2D, MZD that was tested as its hydrochloride salt, strongly affected PMA-stimulated TXB_2 _generation (IC_50 _= 0.23 μM). MZD 10 μM reduced TXB_2 _release to 18.8 % of control (MZD vs. vehicle, respectively 611 ± 342 vs. 3,279 ± 281 pg of TXB_2 _per 200,000 microglia per 70 min, P < 0.01, n = 3). Furthermore, as shown in Fig. 3D, MZD also strongly affected PMA-stimulated O_2_^- ^generation. MZD 0.1 μM reduced O_2_^- ^release to 14 % of control (MZD vs. vehicle, respectively 0.7 ± 0.7 vs. 10.8 ± 0.6 nmol of O_2_^- ^per 200,000 microglia per 70 min, P < 0.01, n = 3).

In contrast, as shown in Fig. 2D the effect of MZD on OPZ-stimulated TXB_2 _generation was limited. MZD 10 μM reduced TXB_2 _generation to 79.9% of control (MZD vs. vehicle, respectively 3,613 ± 469 vs. 4,504 ± 308 pg of TXB_2 _per 200,000 microglia per 70 min, P > 0.05, n = 2). Furthermore, as shown in Fig. 3D, MZD did not affect OPZ-stimulated O_2_^- ^generation even at 10 μM.

As shown in Fig. 2D and 3D, and in contrast to MZB and C, MZD was very cytotoxic to microglia when PMA was used as an agonist to trigger O_2_^- ^and TXB_2 _release: MZD at 0.1 μM caused 61.5 ± 13 % of total LDH released by 0.1 % Triton X-100 treated-microglia (n = 5, P < 0.05). In contrast to the limited effect of MZD on OPZ-stimulated microglia, the data suggests that the reduction of PMA-stimulated O_2_^- ^and TXB_2 _generation resulted from both pharmacological and toxic effects of MZD on LPS-activated microglia cells.

### Effect of manzamine E on LPS-activated neonatal brain microglia TXB_2_, O_2_^- ^and LDH release

As shown in Fig. 1, MZE differs from MZA in having a saturated ketone functionality in the eight-membered amine ring. As depicted in Fig. 2E, MZE inhibited PMA-stimulated TXB_2 _generation with a maximum 49.4 % inhibition observed at 10 μM (MZE vs. vehicle, respectively 1,614 ± 628 vs. 3,279 ± 281 pg of TXB_2 _per 200,000 microglia per 70 min, P > 0.05, n = 3). Furthermore, as shown in Fig. 3E, MZE inhibited PMA-stimulated O_2_^- ^generation with a maximum 26.3 % inhibition observed at 10 μM (MZE vs. vehicle, respectively 8.5 ± 2.1 vs.10.8 ± 0.6 nmol of O_2_^- ^per 200,000 microglia per 70 min, P > 0.05, n = 3).

As shown in Fig. 2E, MZE had limited effect on OPZ-stimulated TXB_2 _generation, with a maximum 43.1 % inhibition at 10 μM (MZE vs. vehicle, respectively 2,594 ± 646 vs. 4,504 ± 308 pg of TXB_2 _per 200,000 microglia per 70 min, P > 0.05, n = 2). Similarly, as depicted in Fig. 3E, MZE did not affect OPZ-stimulated O_2_^- ^generation even at 10 μM.

As shown in Fig 2E and 3E, cytotoxicity of MZE to microglia measured as LDH release was low even at 10 μM (MZE vs. vehicle, respectively 20 ± 6.7 % vs. 19.8 ± 7.7 % of total LDH released by 0.1 % Triton X-100 treated-microglia, P > 0.05, n = 5).

### Effect of manzamine F on LPS-activated neonatal brain microglia TXB_2_, O_2_^- ^and LDH release

MZF differs from MZA in having a saturated ketone functionality in the eight-membered amine ring and hydroxylation at the C-8 position of the β-carboline ring system (Fig. 1). As shown in Fig. 2F, MZF did not inhibit PMA-stimulated TXB_2 _generation. In the presence of 10 μM MZ, TXB_2 _release was 104.1 ± 24.7 % of control TXB_2 _generation (MZF vs. vehicle, respectively 3,202 ± 1,139 vs. 3,279 ± 281 pg of TXB_2 _per 200,000 microglia per 70 min, P > 0.05, n = 3). Similarly, as shown in Fig. 3F, MZF did not inhibit PMA-stimulated O_2_^- ^release. In the presence of 10 μM MZF, O_2_^- ^release was 113 ± 14.5 % of control O_2_^- ^generation (MZF vs. vehicle, respectively 12.8 ± 1.6 vs. 10.8 ± 0.6 nmol of O_2_^- ^per 200,000 microglia per 70 min, P > 0.05, n = 3).

As shown in Fig. 2F, MZF effect on OPZ-stimulated TXB_2 _generation was weak, with a non-statistically significant 26.2 % inhibition at 10 μM (MZF vs. vehicle, respectively 3,317 ± 121 vs. 4,504 ± 308 pg of TXB_2 _per 200,000 microglia per 70 min, P > 0.05, n = 2). Similarly, as depicted in Fig. 3F, MZF was minimally effective in inhibiting OPZ-stimulated O_2_^- ^generation, only a non-statistically significant 15.7 % inhibition observed at 10 μM.

As shown in Fig. 2F and 3F, cytotoxicity of MZF to neonatal brain microglia measured as LDH release was low even at 10 μM (MZF vs. vehicle, respectively 19 ± 7.2 % vs. 19.8 ± 7.7 % of total LDH released by 0.1 % Triton X-100 treated-microglia, n = 5).

### Effect of manzamine A, B, C, D, E and F on hypoxanthine-xanthine oxidase generated O_2_^-^

**Figure 4:**
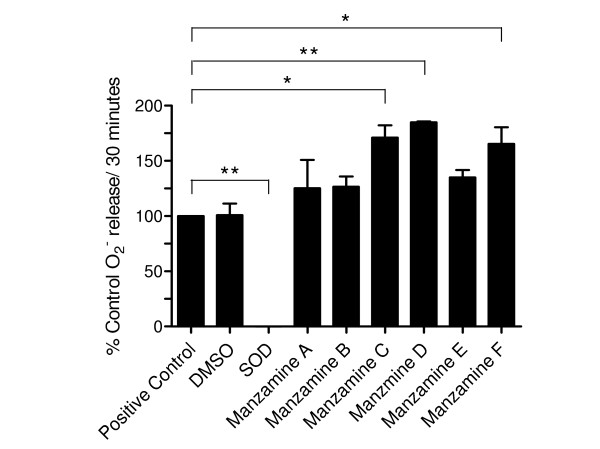
**Differential effect of manzamine A, B, C, D, E and F and superoxide dismutase on hypoxanthine-xanthine oxidase system-generated O_2_^-^. **Cell-free source of O_2_^- ^was generated by a standard hypoxanthine-oxidase system as described in *Materials and Methods*. Data are expressed as O_2_^- ^released (nmoles O_2_^-^/30 min) and correspond to the mean ± SD of 2 representative experiments in duplicate. * P < 0.05, ** P < 0.01 versus control.

In order to determine a potential scavenging effect of MZA, B, C, D, E and F on O_2_^-^, a standard hypoxanthine-xanthine oxidase system was used as a cell-free source of O_2_^- ^[21]. As shown in Fig. 4, O_2_^- ^generation by incubation of purified xanthine oxidase with hypoxanthine was abolished by superoxide dismutase. Furthermore, DMSO, the vehicle used to prepare the manzamines, did not affect O_2_^- ^formation (control vs. DMSO, respectively 12.3 ± 2.3 vs. 12 ± 1.1 nmoles/30 min, n = 2, P > 0.05). Similarly, MZA, B, and E did not significantly affect O_2_^- ^generation (MZA, B, E vs. control, respectively, 15.3 ± 4.4, 15.3 ± 2.4, 16.3 ± 2.2 vs. 12 ± 1.1 nmoles/30 min, n = 2, P > 0.05). In contrast, MZC, D and F appeared to enhance O_2_^- ^formation (MZC, D, F vs. control, respectively 20.7 ± 3.1, 22.2 ± 2, 20.1 ± 3.6 vs. 12 ± 1.1 nmoles/30 min, n = 2, P < 0.05 (MZC, F), P < 0.01 MZD). Thus we conclude that the inhibition of either PMA or OZ stimulated-O_2_^- ^release from LPS-activated microglia by the manzamines was not the result of a direct O_2_^- ^scavenging effect.

## Discussion

The important role of neuroinflammation and glial activation in the pathogenesis of brain disorders has progressively been established [1,2,22]. Because *in vitro *LPS-activated microglia appear to mimic the functions of activated microglia found in neuroinflammatory conditions *in vivo *[2,23], we used LPS-activated rat microglia as a relevant *in vitro *paradigm to search for marine natural products that may modulate the enhanced release of TXB_2 _and O_2_^- ^from activated microglia [24]. Using this *in vitro *model we have previously communicated that MZA, a secondary metabolite isolated from the Okinawan marine sponge *Haliclona *sp[10], inhibited TXB_2 _and O_2_^- ^generation by microglia [18]. The current study extends our initial observations, and reports a structure-activity relationship study with manzamines A, B, C, D, E and F on both PMA and OPZ-stimulated release of TXB_2 _and O_2_^- ^from LPS-activated rat neonatal microglia.

Members of the eicosanoid family (i.e. prostaglandins, leukotrienes and thromboxanes) are important mediators of inflammation that would appear to be play a causative role in the pathogenesis of several CNS disorders [25-27]. Increased levels of eicosanoids have been observed in neurodegenerative disorders such as amyotrophic lateral sclerosis [28], multiple sclerosis [29], ischemia and seizures [30], prion diseases [31], human immunodeficiency virus-associated dementia [32] and Alzheimer's disease [33]. Following the seminal observation that microglia release eicosanoids [34], numerous studies have increasingly supported the notion that activated brain macrophages may be the main source of both prostaglandins [33,35-39] and thromboxanes [23,37,39,40] in these neurodegenerative diseases. Thus modulation of microglia enhanced prostanoid synthesis has been investigated as a potential drug therapeutic approach for intervention in neuroinflammatory disorders of the CNS [36,41,42]. One possible approach to diminish enhanced eicosanoid production has been to search for inhibitors of signal transduction pathways involved in eicosanoid synthesis in activated microglia [42]. In our study, we used PMA and OPZ, agonists known to activate p44/42 and p38 mitogen-activated protein kinases in microglia [43], to target distinct signal transduction pathways that cause TXB_2 _release in rat neonatal microglia activated by an *in vitro *exposure to 0.3 ng/mL of LPS for 17 hours [23]. As shown in Fig. 2, the 6 marine-derived manzamine analogs attenuated PMA-stimulated TXB_2 _generation differentially and in the following order of decreasing potency: MZA>MZD>MZB >MZC>MZE and F. In contrast, the manzamine analogs inhibited OPZ-stimulated TXB_2 _generation with reduced potency: MZB>MZA>MZC>MZD, MZE and MZF. Thus, with the exception of MZB and MZC which modulated both PMA and OPZ-stimulated TXB_2 _release with similar potency, MZA, MZD, MZE and MZF inhibited TXB_2 _release with lower potency when OPZ was used as an agonist.

It is interesting to compare our differential results with the MZA, B, C, D, E, and F with those reported for other agents that have been shown to modulate microglia eicosanoid release by targeting the cyclooxygenase I and II enzymes which are expressed in activated rat and human microglia [35,44]. PGE_2 _and TXB_2 _synthesis that occurs concomitantly with LPS-induced activation of rat [38,45,46] and human microglia [35], has been shown to be attenuated by nonsteroidal anti-inflammatory drugs (NSAIDs) with differing activities towards the two isoforms of COX. Thus, LPS-induced microglia PGE_2 _synthesis was reduced by COX-1 inhibitors: acetylsalicylic acid (aspirin) (IC_50 _= 3.12–10 μM) [38,46], flurbiprofen (apparent IC_50 _= 100 nM) [45] and indomethacin (apparent IC_50 _= 1 nM) [38], and the COX-2 inhibitor NS-398 (apparent IC_50 _= 1–5 nM) [38]. Even though NSAIDs have been reported to attenuate neurotoxicity *in vitro *[47] and neuroinflammation in animal models [48-50], an important caveat is the fact that determining the best NSAIDs for clinical neurodegenerative disease management appears to remain a matter of considerable debate in view of their well known adverse effects [51-53]. Thus, although the molecular mechanism by which the manzamines inhibit TXB_2 _release in LPS-activated cells remains currently undetermined, MZA inhibited PMA-stimulated eicosanoid generation *in vitro *with potency similar to that of the COX-1 inhibitor indomethacin [38], potency that was higher than that of other NSAIDs that have been reported to modulate enhanced eicosanoid release in both activated rat and human microglia [35,38,45,46].

The involvement of reactive oxygen species (ROS) has been documented in CNS pathologies, such as Parkinson's disease, Alzheimer's disease, Huntington's disease, Down's syndrome, cerebral ischemia and reperfusion, amyotrophic lateral sclerosis, multiple sclerosis and meningitis [54]. Prolonged exposure to ROS may potentially damage neurons, particularly their synapses [55] as well as oligodendrocytes, the myelin producing cell of the CNS [56] by overriding normal CNS antioxidant defense mechanisms, e.g. superoxide dismutase, catalase, glutathione-S-transferase, glutathione peroxidase, permanently affecting cellular function [57]. Thus the mechanism of ROS generation by CNS leukocytes, i.e. infiltrating neutrophils and monocytes as well as resident microglia production of O_2_^-^, hydrogen peroxide and nitric oxide in CNS, has received considerable attention since the mid-1980s [2]. In fact, during the past 18 years, numerous research groups have shown that O_2_^- ^may be generated by microglia isolated from rat, mice, hamsters, dogs, swine and humans, when stimulated with a variety of agonists such as phorbol ester, opsonized zymosan, calcium ionophore, antiviral antibodies, antibody-coated red blood cells and myelin (reviewed in [2]). We and others have hypothesized that rather than scavenge ROS with antioxidants, the modulation of the signal transduction mechanism leading to microglia ROS generation might be putatively a better therapeutic strategy to turn off or reduce ROS generation that could lead to neuronal injury [2,58,59]. As depicted in Fig. 3, the manzamine analogs attenuated PMA-stimulated O_2_^- ^generation in the following order of decreasing potency: MZD>MZA>MZB>MZC>MZE and MZF. In contrast, and similarly to their weaker effect on OPZ-stimulated TXB_2 _generation, all the manzamine analogs modulated OPZ-stimulated O_2_^- ^generation with lower potency: MZB>MZC>MZA, MZD, MZE and MZF. Interestingly, as shown in Fig. 4, the effect of MZD, A, B and C on PMA-stimulated O_2_^- ^formation was not the result of any detectable scavenging O_2_^-^, because none of the manzamines inhibited a standard hypoxanthine-xanthine oxidase system that was used as a cell-free source of O_2_^-^. Thus, although the exact mechanism by which the manzamines modulate O_2_^- ^release by microglia remains currently undetermined, we have demonstrated that these compounds clearly modulate the signal transduction pathway that PMA triggers in microglia and ultimately leads to O_2_^- ^generation.

It is interesting to compare our differential results with the MZD, A, B and C with those reported for other agents that modulate PMA-stimulated O_2_^- ^generation in microglia. Interestingly, MZD, A, B and C demonstrated higher potency than three clinically available agents shown to inhibit PMA-stimulated O_2_^- ^generation: propentofylline, a selective phosphodiesterase inhibitor (IC_50 _>100 μM) [60], cabergoline, a potent and selective agonist of D2-dopamine receptors (IC_50 _>100 μM) [59], and nicergoline, an ergoline derivative used for cerebrovascular diseases (IC_50 _= 10–15 μM) [58]. It is noteworthy that these agents have been proposed to confer protective effects against neurodegenerative diseases which may involve O_2_^- ^release by activated rat microglia.

In order to determine if the effect of the manzamines on PMA or OPZ-triggered TXB_2 _and O_2_^- ^generation was either pharmacological or toxic, we investigated LDH release from LPS-activated rat neonatal microglia. LDH has extensively been used as a marker for cell cytotoxicity [61]. The results from our investigation appear to demonstrate that the manzamine analogs clearly differed in their effect on LDH release from LPS-primed microglia: MZA, MZE and MZF generated less than 50% of maximal LDH release at 10 μM, and thus were the least toxic analogs; MZB and MZC induced greater than 50% of LDH release at 10 μM; while MZD showed greater than 50% of LDH release at 0.1 μM when PMA rather than OPZ was used as an agonist, thus its toxicity contrasted with the other analogs tested. Thus even though MZD inhibited PMA-stimulated O_2_^- ^generation with slightly higher potency than MZA, because MZD caused concomitant LDH release at low concentrations (0.1 μM), the nature of the inhibitory effect on PMA-triggered O_2_^- ^and TXB_2 _release, either toxic or pharmacological, remains currently unresolved. In summary, the *in vitro *studies described herein suggest that MZA is the most potent manzamine analog of the series investigated because both PMA-stimulated O_2_^- ^and TXB_2 _were potently inhibited with the *lowest *concomitant release of LDH.

It is of interest to consider the results of our structure-activity relationship (SAR) study with the manzamines, alkaloids characterized by a complex heterocyclic ring system attached to the C-1 of the β-carboline moiety. From the SAR perspective, the potent effect of MZA and D hydrochloride salts on PMA-stimulated O_2_^- ^and TXB_2 _suggests that the solubility or ionic forms are contributing factors to their bioactivity. Furthermore, the fused 13-membered macrocyclic and octahydroisoquinoline ring system, and any substitutions in these rings would appear to be less important for their *in vitro *activity. Finally, changes such as saturation or oxidation of the β carboline or the 8-membered amine ring tended to decrease bioactivity in both O_2_^- ^and TXB_2 _assays.

Taken together, our current data demonstrates that the most potent and least toxic manzamine analog, namely MZA, was less effective in attenuating O_2_^- ^and TXB_2 _from LPS-activated microglia when the triggering agonist was OPZ rather than PMA. Similar differential effects between PMA and OPZ-triggered signaling have been observed with other natural products [62]. Furthermore, the current data suggest the following on the as yet undefined mechanism of action of MZA: Firstly, that the MZA molecular target plays a critical role in O_2_^- ^and TXB_2 _generation initiated by PMA upon binding to PKC [63,64] and activation of the p44/42 mitogen-activated protein kinase signaling pathway [43]; Secondly, that the MZA molecular target probably plays a less critical role in O_2_^- ^and TXB_2 _release elicited by OPZ, a ligand of the microglial cell surface complement receptor 3 shown to activate the p38 mitogen-activated protein kinase signaling pathway [43]. Studies to determine which element is targeted by MZA in the p38 and/or p44/42 mitogen-activated protein kinase pathways in LPS-activated rat microglia are currently underway in our laboratory.

## Conclusion

Our present results provide the first experimental evidence to support the hypothesis that the marine-derived β-carboline alkaloid manzamines differentially modulate both O_2_^- ^and TXB_2 _generated by *E. coli *LPS-activated rat neonatal microglia. Additional conclusions are the following: Firstly, SAR studies demonstrated that at *in vitro *concentrations that were non-toxic to *E. coli *LPS-activated rat neonatal microglia, MZA was the most potent inhibitor of O_2_^- ^and TXB_2. _Secondly, although the mechanism by which MZA inhibited PMA-stimulated TXB_2 _generation *in vitro *is as yet unclear, its potency was similar to the COX-1 inhibitor indomethacin [38], and thus higher than other NSAIDs reported to modulate enhanced eicosanoid release in both activated rat and human microglia [35,38,45,46]. Thirdly, although the mechanism by which MZA inhibited PMA-stimulated O_2_^-^generation *in vitro *remains undetermined, MZA was more potent than propentofylline, a selective phosphodiesterase inhibitor, cabergoline, a potent and selective agonist of D2-dopamine receptors and nicergoline, an ergoline derivative used for cerebrovascular diseases, compounds which have been proposed to confer protective effects against neurodegenerative diseases by affecting O_2_^- ^release by activated rat microglia. Fourthly, SAR studies which demonstrated that the ionic forms are a contributing factor to the bioactivity of the complex manzamine heterocyclic ring system attached to a β-carboline moiety may explain the potent effect of MZA and D hydrochloride salts on PMA-stimulated O_2_^- ^and TXB_2_. Interestingly, the fused 13-membered macrocyclic amine and octahydroisoquinoline ring system, as well as substitutions in these rings appeared to be a non-factor for the *in vitro *activity of the manzamines. Finally, the reported pharmacokinetic properties and the lack of significant *in vivo *toxicity [14] of MZA, a β-carboline alkaloid whose complete synthesis has been reported [11], would suggest that MZA is a prime candidate for further investigation of its potential utility as a pharmacophore from which new and novel therapeutic agents for neuroinflammatory diseases might be developed.

## Methods

### Reagents

LPS B (*Escherichia coli *026:B6) was obtained from Difco Laboratories (Detroit, MI); Wright Giemsa stain (modified), ferricytochrome c type III (from horse heart) (FCC), superoxide dismutase (from bovine liver), phorbol 12-myristate 13-acetate (PMA), zymosan and dimethyl sulphoxide (DMSO) were obtained from Sigma Chemical Co. (St. Louis, MO). PMA was maintained at -80°C as a 10 mM stock solution in DMSO. Opsonized zymosan (OPZ) was maintained at -20°C in a stock solution of 15 mg/ml in PBS and prepared as described [65]. Dulbecco's modified Eagle medium (DMEM) with high glucose (4,500 mg/l), Hank's balanced salt solution (HBSS), penicillin (P), streptomycin (S), trypsin (0.25%)-EDTA (1 mM) and trypan blue were purchased from GIBCO-BRL (Grand Island, NY); certified heat-inactivated fetal bovine serum (FBS) was obtained from Hyclone (Logan, UT); a LPS stock of 1 mg/ml was prepared in a 0.9% sodium chloride nonpyrogenic solution from Baxter Healthcare Corp. (Toronto, ONT, Canada) and then diluted with DMEM plus 10% FBS plus P and S to the appropriate concentration used in our experiments. Both the LPS stock solution [10 ng/ml] and dilutions were stored at -80°C, thawed prior to each experiment and discarded after use.

### LPS containment

To inactivate extraneous LPS, all glassware and metal spatulas were baked for 4 hours at 180°C. Sterile and LPS-free 75- and 162-cm^2 ^vented cell culture flasks, 24-well flat-bottom culture clusters, 96-well cell culture clusters and disposable serological pipettes were purchased from Costar Corporation (Cambridge, MA), while polystyrene cell culture dishes (60 × 15 mm) were obtained from Corning Glass Works (Corning, NY). Sterile and LPS-free Eppendorf Biopur pipette tips were purchased from Brinkmann Instruments, Inc. (Westbury, NY).

### Manzamines A, B, C, D, E and F

Manzamine A (MZA) was isolated from a marine sponge species of the genus *Haliclona *collected off Manzamo, Okinawa in waters at a depth of 30 m in April 1985 [10]. Manzamine B (MZB), manzamine C (MZC) and manzamine D (MZD), were isolated from a sponge of the genus *Amphimedon *collected by SCUBA off Manzamo, Okinawa [66]. Manzamine E (MZE) and manzamine F (MZF) were isolated from a sponge species of the genus *Xestospongia *collected off the coast of Miyako Island, Okinawa in June 1986 [67]. All manzamines were dissolved in DMSO to prepare a 10 mM stock and stored at -80°C prior to use in the experiments.

### Isolation and culture of rat neonatal microglia

All experiments were performed with adherence to the National Institutes of Health guidelines on the use of experimental animals and with protocols approved by Midwestern University's Research and Animal Care Committee. To isolate rat neonatal microglia, cerebral cortices of 1–2 day-old Sprague-Dawley rats purchased from Harlan (Indianapolis, IN) were surgically removed and placed in cold DMEM + 10% FBS + 120 U/ml P and 12 μg/ml S, the meninges carefully removed, and brain tissue minced and dissociated with trypsin-EDTA at 36°C for 3–5 min. The mixed astroglial cell suspension was plated in either 75- or 162-cm^2 ^vented cell culture flasks with DMEM medium supplemented with 10% FBS + 120 U/ml P + 12 μg/ml S and grown in a humidified 5% CO_2 _incubator at 36°C for 12–14 days. On day 14 and every 3–4 days thereafter, microglia were detached using an orbital shaker (150 rpm, 0.5 hours, 36°C, 5% CO_2_), centrifuged (400 × g, 25 min, 4°C), and microglia number and viability assessed by trypan blue exclusion. Microglia were characterized as described earlier [23]. Depending on the particular experimental protocol (see below), microglia averaging greater than 95% viability were plated in 24-well cell culture clusters, with DMEM supplemented with 10% FBS + 120 U/ml P + 12 μg/ml S, and placed in a humidified 5% CO_2 _incubator at 36°C 18–24 hours prior to the experiments.

### Experimental protocol to study the effect of manzamines A – F on microglia release of TXB_2 _and O_2_^-^

To study the effects of manzamines A, B, C, D, E and F on the generation of TXB_2 _and O_2_^-^, rat neonatal microglia (2 × 10^5 ^cells/24-well cell culture clusters) were treated to the following protocol. Seventeen hours prior to the experiments, microglia cells were treated with LPS (0.3 ng/ml) in a final volume of 1 ml of DMEM supplemented with 10% FBS + 120 U/ml P + 12 μg/ml. Thereafter the media was removed and replaced with 1 ml warm HBSS, one of the manzamines (0.1–10 μM final concentration) or vehicle (DMSO) was added, and the microglia incubated for fifteen minutes in a humidified 5% CO_2 _incubator at 35.9°C. After the fifteen minute preincubation period with either manzamines or vehicle, PMA (1 μM) or OPZ (0.5 mg/mL) was added and microglia incubated for 70 minutes in a humidified 5% CO_2 _incubator at 35.9°C in the presence of the manzamines or vehicle. The final concentration of DMSO did not affect microglia viability or LDH release. O_2_^-^, TXB_2 _and lactate dehydrogenase (LDH) release were assayed as described below.

### Assay for TXB_2_

Following the incubation of LPS-activated microglia with HBSS, manzamines or vehicle as explained above, PMA- (1 μM) or OPZ- (0.5 mg/mL)triggered TXB_2 _generation in the culture supernatants was measured using immunoassays (Cayman Chemical, Ann Arbor, MI) as indicated by the manufacturer's protocol. Results were expressed as pg/mL produced after 70 min of PMA or OPZ stimulation.

### Assay for O_2_^-^

O_2_^- ^generation was determined by the SOD-inhibitable reduction of FCC [23]. Briefly, PMA (1 μM) or OPZ (0.5 mg/mL)- triggered O_2_^- ^release from LPS-activated microglia was measured in the presence of FCC (50 μM) and HBSS, with or without SOD (700 Units) which inhibited >95% of FCC reduction, during the 70 min incubation described above. All experimental treatments were run in triplicate and in a final volume of 1 ml. Changes in FCC absorbance were measured at 550 nm using a Beckman DU-650 spectrophotometer. Differences in the amount of reduced FCC in the presence and absence of SOD were used to determine O_2_^- ^generation and expressed in nmol by employing the molecular extinction coefficient of 21.0 × 10^3 ^M^-1 ^cm^-1^.

### Experimental protocol to study the effect of manzamines on superoxide anion by the hypoxanthine-xanthine oxidase system

A standard hypoxanthine-xanthine oxidase system was used as a cell-free source of O_2_^-^. O_2_^- ^was generated by incubation of purified xanthine oxidase (0.02 Units/ml) with hypoxanthine (1.5 mM) at 37°C [21]. O_2_^- ^formation was assessed spectrophotometrically as the increase in absorbance at 550 nm associated with the SOD (30 U/mL)-inhibitable reduction FCC (50 μM) as described above for rat microglia O_2_^- ^generation and expressed in nmoles/30 minutes.

### Lactate dehydrogenase assay

Lactate dehydrogenase (LDH) release from microglia was determined spectrophotometrically as described elsewhere [9]. Microglia LDH release was expressed as a percentage of total LDH. Total LDH resulted from 0.1% Triton X-100-lysed microglia cells (intracellular LDH) plus LDH released to the extracellular medium.

### Statistical analysis of the data

Data were analyzed with the Prism^® ^software package purchased from GraphPad (San Diego, CA.). One-way analysis of variance followed by Dunnett's test was performed on all sets of data. Manzamine-treated groups were compared with the vehicle-treated group, shown as 0 or control in the corresponding figures. Differences were considered statistically significant at p < 0.05 and reported in each figure legend.

## Abbreviations

CNS, central nervous system; DMEM, Dulbecco's modified Eagle medium; DMSO, dimethyl sulphoxide; FBS, fetal bovine serum certified; FCC, ferricytochrome c type III; HBSS, Hank's balanced salt solution; LPS, lipopolysaccharide; MZA, manzamine A; MZB, manzamine B; MZC, manzamine C; MZD, manzamine D; MZE, manzamine E; MZF, manzamine F; O_2_^-^, superoxide anion; OPZ, opsonized zymosan; P, penicillin; PBS, phosphate buffered saline; PMA, phorbol-12-myristate-13-acetate ; S, streptomycin; SOD, superoxide dismutase; TXB_2_, thromboxane B_2_.

## Authors' contributions

**A.M.S. M. **designed and conducted the experiments described and prepared the manuscript draft.

**M.L.H.**, performed the statistical analysis of the data and prepared Fig. 2, 3 and 4.

**S. M. L. **helped conduct the xanthine oxidase studies with Manzamines A, B, C, D, E and F.

**S.P.G. **supplied pure Manzamines A, B, C, D, E. and F from the Division of Biomedical Marine Research depository, prepared Fig. 1, and contributed to various sections of the manuscript.

**S.A.P. **provided financial support and contributed to various sections of the manuscript.

**S.H.S. **contributed to various sections of the manuscript.

All authors read and approved the manuscript.

## References

[B1] Kreutzberg GW (1996). Microglia: a sensor for pathological events in the CNS. Trends Neurosci.

[B2] Mayer AMS (1998). Therapeutic implications of microglia activation by lipopolysaccharide and reactive oxygen species generation in septic shock and central nervous system pathologies: a review. Medicina (B Aires ).

[B3] Mayer AMS, Hamann MT (2004). Marine pharmacology in 2000: marine compounds with antibacterial, anticoagulant, antifungal, anti-inflammatory, antimalarial, antiplatelet, antituberculosis, and antiviral activities; affecting the cardiovascular, immune, and nervous systems and other miscellaneous mechanisms of action. Mar Biotechnol (NY).

[B4] Mayer AMS, Jacobson PB, Fenical W, Jacobs RS, Glaser KB (1998). Pharmacological characterization of the pseudopterosins: novel anti- inflammatory natural products isolated from the Caribbean soft coral, Pseudopterogorgia elisabethae. Life Sci.

[B5] Mayer AMS, Glaser KB, Jacobs RS (1988). Regulation of eicosanoid biosynthesis in vitro and in vivo by the marine natural product manoalide: a potent inactivator of venom phospholipases. J Pharmacol Exp Ther.

[B6] Mayer AMS, Spitzer JA (1993). Modulation of superoxide anion generation by manoalide, arachidonic acid and staurosporine in liver infiltrated neutrophils in a rat model of endotoxemia. J Pharmacol Exp Ther.

[B7] Mayer AMS, Brenic S, Glaser KB (1996). Pharmacological targeting of signaling pathways in protein kinase C- stimulated superoxide generation in neutrophil-like HL-60 cells: effect of phorbol ester, arachidonic acid and inhibitors of kinase(s), phosphatase(s) and phospholipase A2. J Pharmacol Exp Ther.

[B8] Mayer AMS, Spitzer JA (1994). Modulation of superoxide generation in in vivo lipopolysaccharide- primed Kupffer cells by staurosporine, okadaic acid, manoalide, arachidonic acid, genistein and sodium orthovanadate. J Pharmacol Exp Ther.

[B9] Mayer AMS, Brenic S, Stocker R, Glaser KB (1995). Modulation of superoxide generation in in vivo lipopolysaccharide- primed rat alveolar macrophages by arachidonic acid and inhibitors of protein kinase C, phospholipase A2, protein serine-threonine phosphatase(s), protein tyrosine kinase(s) and phosphatase(s). J Pharmacol Exp Ther.

[B10] Sakai R, Higa T (1986). Manzamine A, a novel antitumor alkaloid from a sponge. J Am Chem Soc.

[B11] Hu JF, Hamann MT, Hill R, Kelly M (2003). The manzamine alkaloids. Alkaloids Chem Biol.

[B12] Ang KK, Holmes MJ, Kara UA (2001). Immune-mediated parasite clearance in mice infected with Plasmodium berghei following treatment with manzamine A. Parasitol Res.

[B13] Edrada RA, Proksch P, Wray V, Witte L, Muller WE, van Soest RW (1996). Four new bioactive manzamine-type alkaloids from the Philippine marine sponge Xestospongia ashmorica. J Nat Prod.

[B14] Ang KKH, Holmes MJ, Higa T, Hamann MT, Kara UAK (2000). In vivo antimalarial activity of the beta-carboline alkaloid manzamine A. Antimicrobial Agents & Chemotherapy.

[B15] Rao KV, Kasanah N, Wahyuono S, Tekwani BL, Schinazi RF, Hamann MT (2004). Three new manzamine alkaloids from a common Indonesian sponge and their activity against infectious and tropical parasitic diseases. J Nat Prod.

[B16] Yousaf M, Hammond NL, Peng J, Wahyuono S, McIntosh KA, Charman WN, Mayer AM, Hamann MT (2004). New manzamine alkaloids from an Indo-Pacific sponge. Pharmacokinetics, oral availability, and the significant activity of several manzamines against HIV-I, AIDS opportunistic infections, and inflammatory diseases. J Med Chem.

[B17] El Sayed KA, Kelly M, Kara UA, Ang KK, Katsuyama I, Dunbar DC, Khan AA, Hamann MT (2001). New manzamine alkaloids with potent activity against infectious diseases. J Am Chem Soc.

[B18] Mayer AMS, Gunasekera SP, Pomponi SA, Sennett SH (2000). Inhibition of LPS-primed rat brain microglia superoxide and thromboxane B2 generation by the marine Manzamines.. Society Neuroscience Abstracts.

[B19] Mayer AMS, Gunasekera SP, Pomponi SA, Sennett SH (2002). Anti-inflammatory uses of Manzamines. U S Patent.

[B20] Mayer AMS, Gunasekera SP, Pomponi SA, Sennett SH (2003). Anti-inflammatory uses of Manzamines. U S Patent.

[B21] Lynch SM, Frei B (1993). Mechanisms of copper- and iron-dependent oxidative modification of human low density lipoprotein. J Lipid Res.

[B22] Streit WJ, Mrak RE, Griffin WS (2004). Microglia and neuroinflammation: a pathological perspective. J Neuroinflammation.

[B23] Mayer AMS, Oh S, Ramsey KH, Jacobson PB, Glaser KB, Romanic AM (1999). Escherichia Coli Lipopolysaccharide potentiation and inhibition of rat neonatal microglia superoxide anion generation: correlation with prior lactic dehydrogenase , nitric oxide, tumor necrosis factor-a, thromboxane B2, and metalloprotease release.. SHOCK.

[B24] Mayer AMS, Oh S, Presto E, Glaser KB, Jacobson PB (1997). LPS-primed rat brain microglia: a convenient in vitro model to search for anti-inflammatory marine natural products. SHOCK.

[B25] Eikelenboom P, Bate C, van Gool WA, Hoozemans JJ, Rozemuller JM, Veerhuis R, Williams A (2002). Neuroinflammation in Alzheimer's disease and prion disease. GLIA.

[B26] Moore AH, O'Banion MK (2002). Neuroinflammation and anti-inflammatory therapy for Alzheimer's disease. Adv Drug Deliv Rev.

[B27] Minghetti L (2004). Cyclooxygenase-2 (COX-2) in inflammatory and degenerative brain diseases. J Neuropathol Exp Neurol.

[B28] McGeer PL, McGeer EG (2002). Inflammatory processes in amyotrophic lateral sclerosis. Muscle Nerve.

[B29] Fretland DJ (1992). Potential role of prostaglandins and leukotrienes in multiple sclerosis and experimental allergic encephalomyelitis. Prostaglandins Leukot Essent Fatty Acids.

[B30] Farooqui AA, Horrocks LA (1991). Excitatory amino acid receptors, neural membrane phospholipid metabolism and neurological disorders. Brain Res Brain Res Rev.

[B31] Williams AE, Van Dam AM, Man AHWK, Berkenbosch F, Eikelenboom P, Fraser H (1994). Cytokines, prostaglandins and lipocortin-1 are present in the brains of scrapie-infected mice. Brain Res.

[B32] Griffin DE, Wesselingh SL, McArthur JC (1994). Elevated central nervous system prostaglandins in human immunodeficiency virus-associated dementia. Ann Neurol.

[B33] Hoozemans JJ, Rozemuller AJ, Janssen I, De Groot CJ, Veerhuis R, Eikelenboom P (2001). Cyclooxygenase expression in microglia and neurons in Alzheimer's disease and control brain. Acta Neuropathol (Berl).

[B34] Gebicke-Haerter PJ, Bauer J, Schobert A, Northoff H (1989). Lipopolysaccharide-free conditions in primary astrocyte cultures allow growth and isolation of microglial cells. J Neurosci.

[B35] Hoozemans JJ, Veerhuis R, Janssen I, van Elk EJ, Rozemuller AJ, Eikelenboom P (2002). The role of cyclo-oxygenase 1 and 2 activity in prostaglandin E(2) secretion by cultured human adult microglia: implications for Alzheimer's disease. Brain Res.

[B36] Minghetti L, Polazzi E, Nicolini A, Creminon C, Levi G (1996). Interferon-gamma and nitric oxide down-regulate lipopolysaccharide- induced prostanoid production in cultured rat microglial cells by inhibiting cyclooxygenase-2 expression. J Neurochem.

[B37] Minghetti L, Levi G (1995). Induction of prostanoid biosynthesis by bacterial lipopolysaccharide and isoproterenol in rat microglial cultures. J Neurochem.

[B38] Greco A, Ajmone-Cat MA, Nicolini A, Sciulli MG, Minghetti L (2003). Paracetamol effectively reduces prostaglandin E2 synthesis in brain macrophages by inhibiting enzymatic activity of cyclooxygenase but not phospholipase and prostaglandin E synthase. J Neurosci Res.

[B39] Slepko N, Minghetti L, Polazzi E, Nicolini A, Levi G (1997). Reorientation of prostanoid production accompanies "activation" of adult microglial cells in culture. J Neurosci Res.

[B40] Giulian D, Corpuz M, Richmond B, Wendt E, Hall ER (1996). Activated microglia are the principal glial source of thromboxane in the central nervous system. NEUROCHEM INT.

[B41] Aldskogius H (2001). Regulation of microglia - potential new drug targets in the CNS. Expert Opin Ther Targets.

[B42] Minghetti L, Levi G (1998). Microglia as effector cells in brain damage and repair: focus on prostanoids and nitric oxide. Prog Neurobiol.

[B43] Koistinaho M, Koistinaho J (2002). Role of p38 and p44/42 mitogen-activated protein kinases in microglia. GLIA.

[B44] Minghetti L, Polazzi E, Nicolini A, Creminon C, Levi G (1997). Up-regulation of cyclooxygenase-2 expression in cultured microglia by prostaglandin E2, cyclic AMP and non-steroidal anti-inflammatory drugs. Eur J Neurosci.

[B45] Ajmone-Cat MA, Nicolini A, Minghetti L (2001). Differential effects of the nonsteroidal antiinflammatory drug flurbiprofen and its nitric oxide-releasing derivative, nitroflurbiprofen, on prostaglandin E(2), interleukin-1beta, and nitric oxide synthesis by activated microglia. J Neurosci Res.

[B46] Fiebich BL, Lieb K, Hull M, Aicher B, van Ryn J, Pairet M, Engelhardt G (2000). Effects of caffeine and paracetamol alone or in combination with acetylsalicylic acid on prostaglandin E(2) synthesis in rat microglial cells. NEUROPHARMACOLOGY.

[B47] Klegeris A, Walker DG, McGeer PL (1999). Toxicity of human THP-1 monocytic cells towards neuron-like cells is reduced by non-steroidal anti-inflammatory drugs (NSAIDs). NEUROPHARMACOLOGY.

[B48] Pompl PN, Ho L, Bianchi M, McManus T, Qin W, Pasinetti GM (2003). A therapeutic role for cyclooxygenase-2 inhibitors in a transgenic mouse model of amyotrophic lateral sclerosis. FASEB J.

[B49] Mohanakumar KP, Muralikrishnan D, Thomas B (2000). Neuroprotection by sodium salicylate against 1-methyl-4-phenyl-1,2,3, 6-tetrahydropyridine-induced neurotoxicity. Brain Res.

[B50] Lim GP, Yang F, Chu T, Chen P, Beech W, Teter B, Tran T, Ubeda O, Ashe KH, Frautschy SA, Cole GM (2000). Ibuprofen suppresses plaque pathology and inflammation in a mouse model for Alzheimer's disease. J Neurosci.

[B51] McGeer PL, Schulzer M, McGeer EG (1996). Arthritis and anti-inflammatory agents as possible protective factors for Alzheimer's disease: a review of 17 epidemiologic studies. NEUROLOGY.

[B52] Pasinetti GM (2002). Cyclooxygenase as a target for the antiamyloidogenic activities of nonsteroidal anti-inflammatory drugs in Alzheimer's disease. Neurosignals.

[B53] Hoozemans JJ, Veerhuis R, Rozemuller AJ, Eikelenboom P (2003). Non-steroidal anti-inflammatory drugs and cyclooxygenase in Alzheimer's disease. Curr Drug Targets.

[B54] Halliwell B (2001). Role of free radicals in the neurodegenerative diseases: therapeutic implications for antioxidant treatment. Drugs Aging.

[B55] Colton CA, Fagni L, Gilbert D (1989). The action of hydrogen peroxide on paired pulse and long-term potentiation in the hippocampus. Free Radic Biol Med.

[B56] Griot C, Vandevelde M, Richard A, Peterhans E, Stocker R (1990). Selective degeneration of oligodendrocytes mediated by reactive oxygen species. Free Radic Res Commun.

[B57] Fridovich I (1986). Superoxide dismutases. Adv Enzymol Relat Areas Mol Biol.

[B58] Yoshida T, Tanaka M, Okamoto K (2001). Inhibitory effect of nicergoline on superoxide generation by activated rat microglias measured using a simple chemiluminescence method. Neurosci Lett.

[B59] Yoshida T, Tanaka M, Suzuki Y, Sohmiya M, Okamoto K (2002). Antioxidant properties of cabergoline: inhibition of brain auto-oxidation and superoxide anion production of microglial cells in rats. Neurosci Lett.

[B60] Si Q, Nakamura Y, Ogata T, Kataoka K, Schubert P (1998). Differential regulation of microglial activation by propentofylline via cAMP signaling. Brain Res.

[B61] Morgenstern S, Flor R, Kessler G, Klein B (1966). Automated determination of NAD-coupled enzymes.  Determination of lactic dehydrogenase.. Anal Biochem.

[B62] Nunez CV, Zacheu FM, Pinto E, Roque NF, Colepicolo P, Brigagao MR (2003). Sesquiterpene lactone from Wunderlichia crulsiana inhibits the respiratory burst of leukocytes triggered by distinct biochemical pathways. Life Sci.

[B63] Giulian D, Baker TJ (1986). Characterization of ameboid microglia isolated from developing mammalian brain. J Neurosci.

[B64] Colton CA, Gilbert DL (1987). Production of superoxide anions by a CNS macrophage, the microglia. FEBS Lett.

[B65] Johnston RBJ (1984). Measurement of O2- secreted by monocytes and macrophages. Methods Enzymol.

[B66] Sakai R, Kohmoto S, Higa T, Jefford CW, Bernardinelli G (1987). Manzamine B and C, two novel alkaloids from the sponge Haliclona sp.. Tetrahedron Lett.

[B67] Ichiba T, Sakai R, Kohmoto SSG (1988). New manzamine alkaloids from a sponge of the genus Xestospongia. Tetrahedron Lett.

